# Preliminary Effects of Hyperbaric Oxygen Therapy on Hair Follicle Characteristics in Healthy Subjects

**DOI:** 10.3390/bioengineering13020240

**Published:** 2026-02-19

**Authors:** Hee Young Lee, Ji Yong Lee, Seung Chan Kim, Yoonsuk Lee

**Affiliations:** 1 Department of Emergency Medicine, Yonsei University Wonju College of Medicine, Wonju 26426, Gangwon, Republic of Korea; hylee3971@yonsei.ac.kr; 2 Research Institute of Hyperbaric Medicine and Science, Yonsei University, Wonju 26426, Gangwon, Republic of Korea; 3 Department of Anatomy, Kosin University College of Medicine, Busan 49267, Republic of Korea; jylee740218@gmail.com; 4 Department of Plastic Surgery, Ieul Plastic Surgery Clinic, Seoul 06576, Republic of Korea; chowsaeng@gmail.com

**Keywords:** hyperbaric oxygen therapy, hair follicle, hair growth, phototrichogram, tissue regeneration

## Abstract

**Background**: Hyperbaric oxygen therapy (HBOT) has regenerative effects in various tissues, but its impact on hair follicles is unclear. This preliminary study evaluated HBOT-induced changes in hair and scalp characteristics in healthy adults. **Methods**: Nine healthy volunteers completed 50 HBOT sessions over three months (2.0 ATA, 100% oxygen, 90 min per session). Objective assessments included follicle density, hairs per follicle, hair volume, and shaft thickness using the Becon phototrichogram system. Subjective evaluations were conducted via a 7-point Likert questionnaire on scalp appearance, hair density, thickness, growth, and shedding. Pre- and post-treatment data were compared using paired statistical tests. **Results**: Positive trends were observed in follicle density (61.3→66.8 counts/cm^2^), hairs per follicle (1.24→1.33), and hair volume (24.9→27.7%), though not statistically significant. Hair shaft thickness decreased significantly (0.18→0.10 mm, *p* = 0.011), consistent with early anagen-phase regrowth. Subjective assessments showed significant improvements across all domains (*p* < 0.05). Scalp imaging visually supported these findings. **Conclusions**: HBOT may enhance hair follicle activation and scalp health in healthy adults. These preliminary findings justify further controlled studies to explore HBOT as a non-pharmacological approach to hair regeneration.

## 1. Introduction

Hair and hair follicles are biologically significant structures that play a crucial role in thermoregulation, protection, and sensory function. Although hair loss is not a life-threatening condition, it can have profound psychological and emotional consequences, significantly affecting an individual’s quality of life [1,2]. Alopecia can occur regardless of age or gender, and the incidence of depression among patients with hair loss has been reported to be notably high. The formation of hair begins with the development of the initial hair follicles from the epidermis. Hair growth follows a well-defined cycle consisting of the anagen (growth), catagen (regression), telogen (resting), and subsequent return to anagen phases. Disruption of this cycle may result in various types of hair loss, with causes including hormonal imbalances, immune system dysfunction, nutritional deficiencies, certain medications, childbirth, high fever, surgery, or psychological stress [3]. Among these, androgenetic alopecia—commonly known as male-pattern hair loss—is the most prevalent form. It is primarily driven by the action of dihydrotestosterone (DHT), a metabolite of testosterone, which causes miniaturization of hair follicles. Currently, U.S. Food and Drug Administration (FDA)-approved treatments include oral finasteride and topical minoxidil [4,5]. Other types of hair loss include telogen effluvium, which often occurs after childbirth or extreme dieting, and alopecia areata, which is related to autoimmune dysfunction. These conditions often improve upon the resolution of the underlying causes. However, senile alopecia associated with aging lacks effective treatments and is generally managed through lifestyle modifications as it stems from a diminished regenerative capacity of hair follicles.

Hyperbaric oxygen therapy (HBOT) involves inhaling 100% oxygen at pressures exceeding 1.4 absolute atmospheres (ATA) [6,7]. HBOT was initially introduced in the 1960s to treat acute carbon monoxide poisoning in Korea. HBOT is recognized for its clinical benefits, including wound healing, radiation injury, and chronic infections, resulting from its effects on tissue oxygenation, inflammation modulation, and cell regeneration. In Korea, the widespread installation of HBOT chambers in the 1980s declined over time due to shifts in the source of the heating fuel and limited clinical application beyond carbon monoxide intoxication [8]. The Undersea and Hyperbaric Medical Society (UHMS) recommends therapeutic administration of hyperbaric oxygen therapy (HBOT) at pressures between 2.0 and 3.0 Atmospheres Absolute (ATA) for 90 to 120 min for most medical indications.

Despite the proven benefits of HBOT in tissue regeneration and inflammation control, few studies have examined its potential effects on hair follicle physiology. Recent investigations have explored HBOT primarily as an adjunctive intervention in the context of hair transplantation surgery, focusing on postoperative recovery and graft survival in patients with alopecia [9,10]. Emerging evidence suggests that the secondary mechanisms of HBOT, such as anti-hypoxia effects, capillary regeneration, and mitigation of ischemia–reperfusion injury, may extend benefits to other tissues, including the scalp [11]. Experimental evidence further supports the biological plausibility of oxygen modulation in hair follicle regulation. Kato et al. reported that ischemic conditions adversely affected hair growth and cycling, whereas hyperoxygenation was associated with modulation of hair cycle dynamics and enhanced follicular activity [12]. Moreover, oxygen-enriched environments have been shown to influence the expression of growth factors closely associated with hair follicle maintenance and regeneration, including vascular endothelial growth factor (VEGF), transforming growth factor-β (TGF-β), and platelet-derived growth factor (PDGF). These mediators are known to contribute to angiogenesis, dermal papilla signaling, and regulation of the anagen phase. Recent regenerative medicine literature has further emphasized the importance of microvascular support and growth factor-mediated signaling pathways in sustaining hair follicle vitality [13]. Collectively, these findings suggest that oxygen-based interventions, including hyperbaric or normobaric oxygen exposure, may influence hair follicle physiology through modulation of the perifollicular microenvironment. These mechanisms have demonstrated efficacy in treating spinal cord injuries and compromised skin grafts or flaps, suggesting a potential role in supporting hair follicle health and promoting hair regrowth [14]. However, these prior studies evaluated HBOT in conjunction with surgical intervention rather than examining the isolated effects of HBOT exposure itself. Moreover, limited research has investigated the impact of HBOT on hair-related parameters in non-surgical settings, particularly among otherwise healthy individuals without clinically diagnosed alopecia. As a result, the independent contribution of controlled hyperbaric oxygen exposure to intrinsic hair follicle dynamics remains insufficiently characterized. In contrast, the present study was not designed to assess surgical outcomes or graft viability, but instead to explore whether controlled HBOT exposure alone, in the absence of surgical manipulation, is associated with measurable changes in intrinsic hair follicle-related parameters in healthy individuals.

Given this background, the present study aims to evaluate the effects of hyperbaric oxygen therapy on hair follicles and hair characteristics in healthy adult subjects. By analyzing pre- and post-therapy morphological changes, this research seeks to explore the regenerative potential of HBOT in a novel domain and assess its implications for both medical and esthetic applications. By integrating objective phototrichogram-based measurements with structured subjective assessments, this study seeks to address this translational gap and provide preliminary data on HBOT exposure independent of procedural confounders. Rather than establishing therapeutic efficacy, this exploratory investigation seeks to generate preliminary observational data that may inform future controlled studies in clinical hair loss populations.

## 2. Materials and Methods

### 2.1. Study Design and Ethical Approval

This study is a prospective, preliminary clinical trial conducted over three months at two HBOT centers, involving a total of 50 hyperbaric oxygen therapy (HBOT) sessions in nine healthy adult participants. It was designed to observe and analyze changes in hair follicle-related and hair characteristics following HBOT exposure. Initially, 16 volunteers were screened for eligibility; however, three individuals were excluded during the screening process (one due to claustrophobia and two due to personal reasons), and four participants were lost to follow-up before completing the intervention, resulting in a final cohort of nine participants. This study hypothesized that the secondary mechanisms of hyperbaric oxygen therapy, such as the anti-hypoxic effect and the promotion of capillary regeneration, could aid hair follicle cell regeneration by enhancing peripheral oxygen supply and promoting tissue regeneration.

The study was approved by Yonsei University Wonju Severance Christian Hospital Research Ethics Committee (IRB approval number: CR322058), as well as by the Public Institutional Review Board designated by the Ministry of Health and Welfare of Korea (approval number: P01-202311-01-005). Along with IRB approval, it was registered with the Clinical Research Information Service (CRIS) (Trial Registration No.: KCT0011058), which is a primary registry of the WHO International Clinical Trials Registry Platform (ICTRP) and thus ensures international compatibility. This clinical study complied with the International Conference on Harmonization (ICH) Guidelines and the principles of the Declaration of Helsinki and was conducted in accordance with the Korean Good Clinical Practice (KGCP) and related regulations, taking into account the rights and safety of the subjects. All participants provided written informed consent.

### 2.2. Medical Device and HBOT Protocol for Clinical Trials

**Figure 1:**
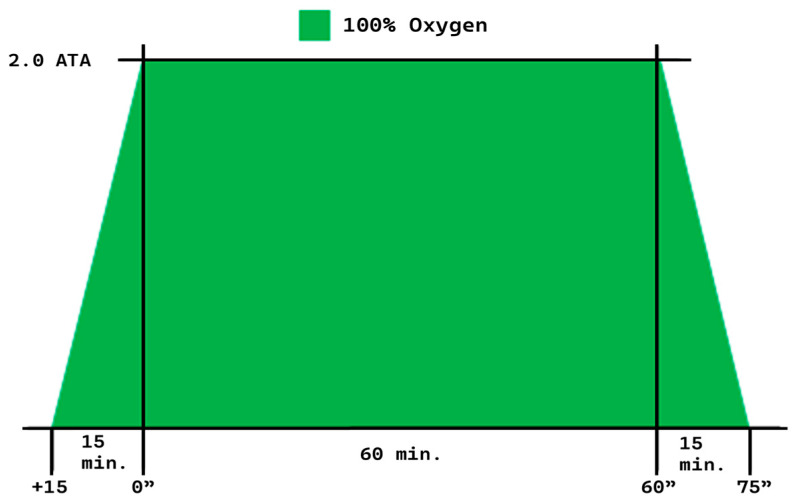
Hyperbaric oxygen therapy protocol.

A hyperbaric oxygen chamber, IBEX LIGHT (IBEX Medical Systems Co., Ltd., Wonju, Republic of Korea), was used to administer hyperbaric oxygen therapy (HBOT). This single-person, medical-grade monoplace chamber is designed for clinical applications. It delivers 100% oxygen at pressures of up to 2 atmospheres absolute (ATA), making it suitable for enhanced tissue oxygenation conditions. We utilized the hyperbaric oxygen treatment protocol number 9, which was established by the US Navy. We modified it by lowering the target pressure to 2.0 ATA, shortening the treatment time, and gradually increasing and decreasing the pressure over 15 min, allowing patients to adapt to the pressure change smoothly (Figure 1) [15].

### 2.3. Phototrichogram Analysis Using the Becon System (Quantitative Evaluation)

The Becon system (Becon AI Scanner, Becon Co., Ltd., Seoul, Republic of Korea) was employed to perform phototrichogram-based scalp analysis. This digital diagnostic tool is equipped with an optical camera and multiple integrated sensors, enabling the real-time capture of high-resolution scalp images at 30 frames per second [16]. The system simultaneously assesses scalp and hair health by measuring parameters such as scalp temperature, moisture levels, and volatile organic compounds (VOCs), which serve as indicators of heat, dryness, odor, and sensitivity. The device wirelessly connects to a dedicated mobile or tablet application, which processes and analyzes the collected data. A high-magnification (20×) lens provides detailed imaging of the scalp surface, while embedded biosensors acquire biological signals that are translated into clinically relevant diagnostic metrics through proprietary software. The Becon system evaluates 11 scalp health parameters, among which four key indicators are directly related to hair loss: follicle density, number of hairs per follicle, hair thickness, and hair volume [17,18].

- Follicle density refers to the number of follicles per square centimeter (cm^2^), automatically assessed using machine learning algorithms. Higher density indicates healthier, fuller hair, while low density suggests thinning.
- Number of hairs per follicle measures how many hairs emerge from a single follicle. The system uses color-coded visualization (e.g., red for one hair, yellow for two, green for three) to aid interpretation. A greater number of hairs per follicle is indicative of healthier hair.
- Hair thickness is automatically calculated across all visible strands within a 1 cm^2^ area and reported as an average in millimeters. Thicker hairs suggest better hair health and coverage.
- Hair volume (coverage) is presented as a percentage, representing the visible amount of hair within a 1 cm^2^ area.

To evaluate the severity of hair loss, the Becon system compares measurements from two regions: a “Healthy Scalp Area” and a “Scalp Concern Area”. Each of the four metrics is compared between the two areas. Based on the cumulative difference in the four metrics, a comprehensive Health Loss Health Score is generated, ranging from 0 to 100. Depending on the degree of deviation, the system categorizes the result as “Healthy (85 points and above),” “Good (65 to 84 points),” “Caution (36 to 64 points),” or “Warning (35 points and below).” Finally, the Becon system enables longitudinal tracking of scalp and hair conditions. It monitors changes in follicle density, hair count per follicle, hair thickness, and overall coverage over time, providing precise visual and quantitative data on improvement or deterioration across treatment sessions.

### 2.4. Questionnaire (Qualitative Evaluation)

Table 1 shows the hair condition (scalp appearance, crown, amount of hair, hair thickness, hair growth rate, and frontal hairline) before and after hyperbaric oxygen therapy was evaluated using a 7-point Likert scale ranging from “very worse (1 point)” to “very improved (7 points).” This questionnaire was developed specifically for the present exploratory study and has not undergone formal psychometric validation. It was used as a pragmatic tool to capture participant-perceived changes; therefore, the subjective findings should be interpreted with appropriate caution.

### 2.5. Study Flow and Protocol Overview

**Figure 2:**
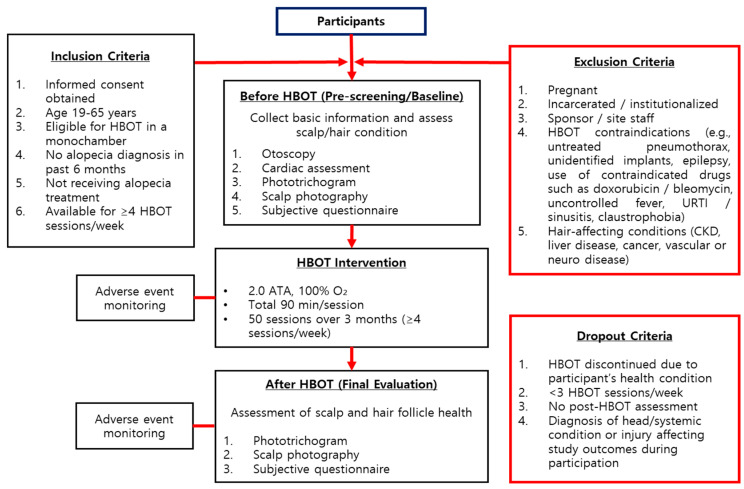
Flowchart of clinical trials.

The overall study protocol was structured into three main phases: pre-screening and baseline assessment, HBOT intervention, and final evaluation (Figure 2). Participants were recruited based on specific inclusion criteria, including adults aged 19 to 65 who were capable of undergoing HBOT in a hyperbaric monochamber, with no history of hair loss diagnosis or treatment in the previous six months. Exclusion criteria included pregnancy, incarceration, institutionalization, contraindications for HBOT, and possession of specific medical devices. During the screening and baseline phases, participants underwent a comprehensive physical examination, a medical history review, and safety assessments, including otoscopic and cardiovascular evaluations. Scalp and hair condition were objectively assessed using the Becon phototrichogram system, and subjective perceptions were recorded through structured questionnaires. The HBOT intervention was administered at 2.0 ATA (atmospheres absolute) with 100% oxygen for 90 min per session (15 min compression, 60 min oxygenation, 15 min decompression) at least four times a week for a total of 50 sessions over three months. All treatments were conducted in a controlled chamber environment, with safety protocols strictly followed, including pre-treatment education on pressure equalization techniques and restrictions on prohibited items. After completing the 3-month intervention, participants underwent the same evaluations as those conducted during the baseline phase to assess changes in hair and scalp condition, subjective perceptions, and to monitor for any adverse effects. This structured flow ensured both clinical safety and methodological consistency throughout the study.

### 2.6. Statistical Analysis

Statistical analyses were conducted using SPSS Statistics (Version 28.0, IBM Corp., Armonk, NY, USA). To evaluate the effects of hyperbaric oxygen therapy (HBOT), appropriate statistical tests were applied according to the type and distribution of each variable. For continuous variables (e.g., follicle density, hair per follicle, hair volume, hair thickness, and total score), normality was assessed using the Shapiro–Wilk test. If the data were normally distributed, a paired t-test was used to compare the means before and after HBOT. When normality was not satisfied, the non-parametric Wilcoxon signed-rank test was employed. For categorical variables (e.g., presence or absence of symptoms or complications), the McNemar test was used to assess changes in paired proportions before and after treatment. Subjective outcomes were evaluated using six self-reported items rated on a 7-point Likert scale (e.g., “The overall appearance of the scalp is improving”, “The crown of the head is growing longer”, “The amount of hair falling out is decreasing”, “The hair is getting thicker”, “The hair growth rate is getting faster”, and “The front hairline is improving”). Given the ordinal nature of these responses, pre- and post-treatment comparisons were conducted using the Wilcoxon signed-rank test. To visually illustrate changes in response patterns, frequency distribution plots were generated. All statistical tests were two-tailed, and a *p*-value of less than 0.05 was considered statistically significant. In addition to *p*-values, effect sizes were calculated to estimate the magnitude of observed changes. For paired comparisons, Cohen’s d was calculated by dividing the mean of the paired differences by the standard deviation of the difference scores (dz) and interpreted as small (0.2), medium (0.5), or large (0.8) effects.

## 3. Results

Table 2 describes the characteristics and medical history of the participants. A total of nine healthy adult participants were enrolled in the study, comprising five females (55.6%) and four males (44.4%). The mean age was 39.3 ± 10.8 years, and the median age was 37 years with an interquartile range (IQR) of 31.5 to 45 years. Age group distribution was as follows: one participant (11.1%) in their 20s, four (44.4%) in their 30s, three (33.3%) in their 40s, none in their 50s, and one (11.1%) in their 60s. All participants were free of known past medical conditions and did not undergo a physical examination, including anthropometric assessment and chest radiography, at the time of data collection.

### 3.1. Objective Evaluation of Hair Condition Before and After HBOT

To complement the subjective assessments, quantitative hair measurements were conducted before and after HBOT. As summarized in Table 3, the overall hair health score showed an increasing trend from 73.11 ± 4.76 point to 75.44 ± 3.88 point, although the change did not reach statistical significance (*p* = 0.098, Cohen’s d = 0.624). A similar upward trend was observed in follicle density, which increased from 61.33 ± 13.00 counts/cm^2^ to 66.78 ± 6.32 counts/cm^2^ (*p* = 0.216, Cohen’s d = 0.447), suggesting a potential enhancement in scalp follicular activity. Furthermore, the average number of hairs per follicle rose from 1.24 ± 0.03 counts to 1.33 ± 0.14 counts (*p* = 0.099, Cohen’s d = 0.620), and hair volume increased from 24.89 ± 3.63% to 27.69 ± 4.87% (*p* = 0.097, Cohen’s d = 0.627). While these changes were not statistically significant, the consistent directional improvements across multiple parameters should be interpreted with caution. Given the absence of a control group and the limited sample size, the study is underpowered to support causal inference, and these findings should be considered exploratory rather than indicative of definitive biological effects. Furthermore, given the small sample size and the multiple statistical comparisons performed, these observed trends may also reflect random variability rather than true underlying biological change. Interestingly, a statistically significant reduction was observed in hair shaft thickness, decreasing from 0.18 ± 0.08 mm to 0.10 ± 0.02 mm (*p* = 0.011, Cohen’s d = 1.093). This finding may reflect early-stage hair regeneration, characterized by the production of thinner, newly growing hair shafts. Taken together, these objective measurements suggest directional changes in hair-related parameters; however, given the limited sample size and reduced statistical power, these findings should be considered exploratory and hypothesis-generating rather than confirmatory.

**Figure 3:**
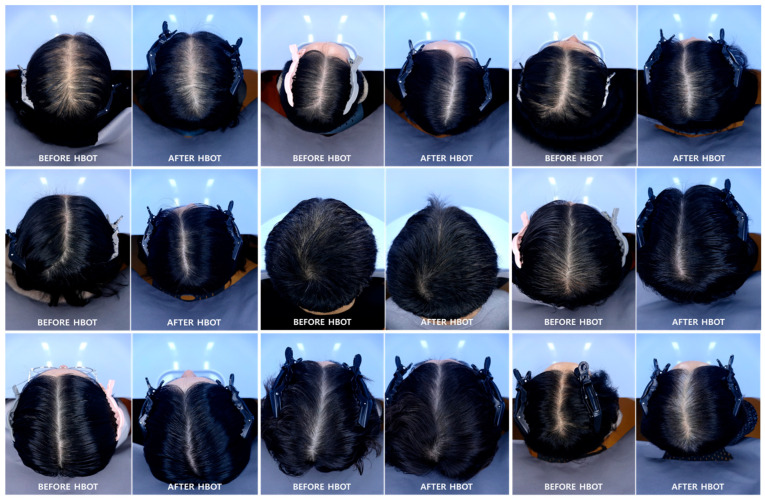
Visual documentation of scalp condition before and after HBOT.

The standardized scalp images were obtained using the Becon imaging system before and after the HBOT sessions to visually corroborate the findings. As shown in Figure 3, a series of top-view photographs illustrates noticeable changes in scalp and hair condition across multiple participants. Consistent lighting and positioning allowed for reliable visual comparison between the pre- and post-treatment states. Post-HBOT images typically show a noticeable improvement in hair density and coverage, particularly in the vertex and crown areas. Several subjects display a reduction in scalp exposure and an increase in darkening or thickening of hair, suggesting improved follicular activity. These changes align with the quantitative trends observed in hair volume, follicle density, and subjective perception, suggesting a possible association between HBOT exposure and changes in hair-related parameters. While visual assessments are inherently qualitative, the consistency of improvement across a broad range of subjects further supports the positive impact of HBOT on scalp health and hair appearance.

### 3.2. Assessment for Improvement in Hair Condition Before and After HBOT

**Figure 4:**
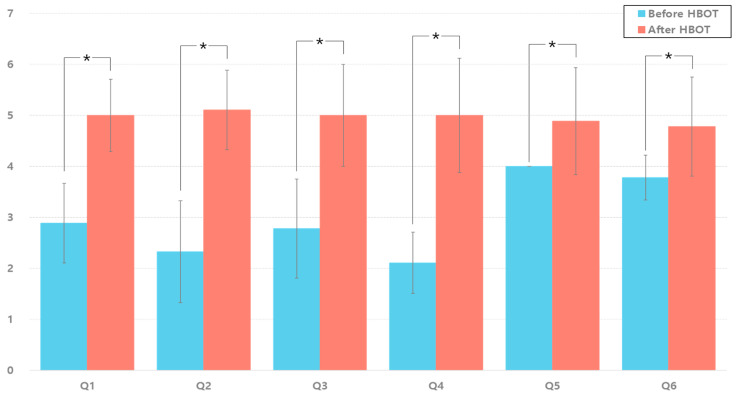
Questionnaire evaluation before and after HBOT for improvement in hair condition.; * indicates a statistically significant difference between pre- and post-HBOT measurements (*p* < 0.05).

To assess subjective improvement in hair condition following hyperbaric oxygen therapy (HBOT), a structured questionnaire comprising six items was administered before and after the intervention. As shown in Figure 4, all questionnaire items demonstrated statistically significant improvement post-HBOT. Participants reported a substantial improvement in the overall appearance of the scalp, with mean scores increasing from 2.89 ± 0.78 to 5.00 ± 0.71 (Z = −2.687, *p* = 0.007). Similarly, perceived hair growth at the crown of the head significantly improved (2.33 ± 1.00 to 5.11 ± 0.78; Z = −2.684, *p* = 0.007). A notable reduction in hair shedding was also observed (2.78 ± 0.97 to 5.00 ± 1.00; Z = −2.536, *p* = 0.011). Moreover, participants reported an increase in hair thickness (2.11 ± 0.60 to 5.00 ± 1.12; Z = −2.699, *p* = 0.007) and hair growth rate (4.00 ± 0.00 to 4.89 ± 1.05; Z = −2.060, *p* = 0.039). Improvement in the anterior hairline was also significant (3.78 ± 0.44 to 4.78 ± 0.97; Z = −2.060, *p* = 0.039). These findings positively contribute to the overall outcomes of this study.

## 4. Discussion

This study investigated the potential regenerative effects of HBOT on hair and scalp health through both objective measurements and structured subjective assessments. Although most objective parameters, including overall hair health score, follicle density, hair volume, and the average number of hairs per follicle, did not reach statistical significance, they exhibited consistent positive trends following HBOT. These directional changes may reflect subtle modulation of the follicular microenvironment, potentially related to improved tissue oxygenation and microvascular support. Hyperoxic exposure has been associated with angiogenic signaling and capillary remodeling in other tissues, suggesting a plausible biological pathway through which HBOT could influence perifollicular conditions. However, given the exploratory design, limited sample size, and absence of a control group, these findings should be interpreted as preliminary and hypothesis-generating rather than confirmatory evidence of efficacy.

Notably, a statistically significant decrease in hair shaft thickness was observed. One possible interpretation is that this finding reflects early anagen-phase regrowth, during which newly emerging vellus or intermediate hairs are typically finer in diameter. However, alternative explanations should also be considered. The observed reduction may be influenced by measurement variability inherent to phototrichogram-based analysis, changes in hair cycle synchronization, or shifts in the proportion of growing versus resting hairs within the sampled area. Given that this was the only objectively significant parameter and the study was conducted in a small cohort without a control group, this finding should be interpreted cautiously and cannot be conclusively attributed to follicular regeneration. These findings are in concordance with prior studies that support the biological plausibility of HBOT in promoting hair regeneration. It demonstrated that normobaric hyperoxia significantly enhanced hair growth and delayed the catagen phase in mice, highlighting the importance of oxygen levels in follicular cycling [12]. HBOT has been shown to upregulate angiogenic factors such as vascular endothelial growth factor (VEGF) and basic fibroblast growth factor (bFGF), both of which contribute to perifollicular vascularization and follicle homeostasis [10,19]. However, it is unlikely that any observed changes in hair follicle dynamics arise from a single isolated molecular pathway. Enhanced vascularity and oxygenation may create a microenvironment theoretically favorable for hair follicle stem cell and dermal papilla activity; however, the present study design does not allow confirmation of this mechanistic relationship [20]. Recent literature emphasizes that hyperoxic exposure triggers system-level adaptations involving neurovascular coupling, metabolic reprogramming, and regenerative signaling cascades rather than linear cause–effect mechanisms [21,22]. In this context, any potential effects of HBOT on hair physiology should be understood as emerging from integrated biological networks rather than from direct follicular stimulation alone.

Subjective assessments in this study revealed statistically significant improvements across all evaluated domains, including perceived hair density, thickness, reduced shedding, and improved anterior hairline. However, these robust subjective improvements contrasted with the largely non-significant objective findings, highlighting a divergence that warrants careful interpretation. These self-reported outcomes are consistent with prior reports suggesting that enhanced oxygenation may influence scalp microcirculation and perceived hair characteristics [23]; however, the present findings were observed in healthy individuals and should not be directly extrapolated to clinical alopecia populations. Although participants reported improvement in the frontal hairline, this observation should be interpreted cautiously. In the absence of controlled comparison and objective confirmation, it remains unclear whether this reflects physiological change or perceptual bias.

Standardized scalp imaging using the Becon system provided visual corroboration of therapeutic effect, revealing apparent increases in hair density and coverage, particularly in the vertex and crown regions [24]. However, these observations were based on photographic comparison and should be interpreted descriptively, as the system is subject to variability in image acquisition and does not provide definitive confirmation of therapeutic efficacy. These visual changes were consistent with both subjective feedback and objective data trends, further supporting a potential association between HBOT exposure and observed changes in scalp and hair-related parameters [25]. However, the use of photographic assessment represents an important source of variability. Despite attempts to maintain consistent imaging conditions, factors such as lighting, camera angle, hair styling, and cosmetic treatments (including hair dyeing) were not fully standardized and may have influenced visual perception of hair density and coverage, particularly in the absence of a control group [26].

Despite the promising nature of these findings, several limitations must be acknowledged. First and foremost, the small sample size (*n* = 9) restricts the statistical power of the study and limits its generalizability. A larger cohort is needed to confirm these preliminary trends and to assess treatment efficacy more robustly. Second, the lack of a control group hinders the ability to attribute observed changes solely to HBOT. Without comparison to a placebo or non-treatment arm, potential placebo effects or confounding factors—such as seasonal hair changes or grooming habits—cannot be ruled out. Third, although the study involved 50 treatment sessions over three months, this duration may have been insufficient to observe the full extent of follicular transformation, particularly the conversion of vellus hairs into terminal hairs. Hair growth is a slow process, and longer follow-up periods are necessary to evaluate the sustainability and progression of treatment effects. In addition, the present study did not assess long-term outcomes beyond the intervention period. Therefore, it remains unclear whether the observed directional trends would persist, stabilize, or diminish over time. Larger-scale longitudinal studies are required to confirm the durability of any potential effects on hair characteristics. Although no serious adverse events were reported during the intervention, repeated HBOT exposure may be associated with mild transient symptoms such as headache or dizziness. Furthermore, theoretical concerns regarding potential physiological overstimulation, including possible alterations in scalp sebum production, should be considered. Future investigations should incorporate systematic safety monitoring to better characterize both short- and long-term tolerability. Fourth, the HBOT protocol used in this study was adapted from the US Navy protocol rather than a dermatologically standardized regimen. This modification, intended to accommodate participant compliance and safety, may limit direct comparability with other HBOT studies and underscores the need for standardized treatment protocols in future trials. Fifth, the discrepancy between subjective improvement and largely non-significant objective findings (except hair shaft thickness) raises important questions. While the consistent positive trends across objective parameters suggest biological response, it is also possible that the subjective benefits were influenced by expectancy effects. Integrating histological analyses, such as scalp biopsies or trichoscopy, in future research could provide more definitive evidence of follicular regeneration at the cellular level. Sixth, while photographs offered a valuable visual tool for evaluating hair condition, the resolution was insufficient to demonstrate individual follicle-level changes. Additionally, hair dyeing status, which was not standardized, may have affected visual perception of hair thickness or color. Future studies should incorporate high-resolution imaging technologies and control for such variables to ensure more accurate visual assessment. Lastly, while no adverse effects (e.g., barotrauma, dizziness, oxygen toxicity) were reported during the intervention period, a formal evaluation and documentation of safety outcomes should be included in future research. Establishing a favorable safety profile is essential for broader clinical application.

Taken together, these limitations highlight the preliminary nature of the current study. Although subjective improvements and directional trends in objective parameters were observed, the small sample size substantially limits inferential strength. Therefore, these findings should be interpreted as preliminary observations that warrant validation in adequately powered controlled studies. Moreover, with only nine participants completing the protocol, the study was likely underpowered to detect small-to-moderate effect sizes. As such, the absence of statistical significance in several parameters may reflect limited power rather than true absence of effect, while observed directional trends should not be overinterpreted as evidence of biological efficacy. Future studies should incorporate larger sample sizes, randomized controlled designs, histological validation, and extended follow-up periods to substantiate these early findings and clarify the clinical utility of HBOT in the field of trichology.

## 5. Conclusions

Hyperbaric oxygen therapy (HBOT) is increasingly being explored across various medical fields, yet its potential applications in trichology remain insufficiently investigated. This study explored the effects of HBOT on hair and follicle health in healthy adults, revealing positive changes supported by both subjective perceptions and objective measures. These preliminary findings suggest that HBOT exposure was associated with changes in scalp and hair-related parameters in healthy adults. However, given the absence of a control group, the exploratory design, and the inclusion of participants without diagnosed alopecia, these results should be interpreted cautiously and should not be considered evidence of therapeutic efficacy in clinical hair loss conditions. Rather, the present findings are hypothesis-generating and may inform future controlled studies in patients with alopecia. While the current study had limitations, including a small sample size, absence of long-term follow-up, and use of a modified HBOT protocol, the observed trends, although derived from a small and underpowered sample, warrant further investigation in larger, adequately powered randomized controlled trials. Future research incorporating standard protocols, histological analysis, and inclusion of patients with alopecia will be essential to clarify the clinical applicability of HBOT in trichology.

## Figures and Tables

**Table 1:** Questionnaire about hair condition before and after HBOT.

Questions	Qualitative Evaluation						
	1	2	3	4	5	6	7
Q1. The overall appearance of the scalp is improving.	□	□	□	□	□	□	□
Q2. The crown of the head is growing longer.	□	□	□	□	□	□	□
Q3. The amount of hair falling out is decreasing.	□	□	□	□	□	□	□
Q4. The hair is getting thicker.	□	□	□	□	□	□	□
Q5. The hair growth rate is getting faster.	□	□	□	□	□	□	□
Q6. The front hairline is improving.	□	□	□	□	□	□	□

**Table 2:** Characteristics and medical history of participants (*n* = 9).

Characteristic	Value
Sex, *n* (%)	
Female	5 (55.6)
Male	4 (44.4)
Age (years)	
Average (mean ± SD)	39.3 ± 10.8
Median ([IQR])	37 [31.5–45]
Age group, *n* (%)	
20s	1 (11.1)
30s	4 (44.4)
40s	3 (33.3)
50s	0 (0)
60s	1 (11.1)
History of past illness, *n* (%)	
None	9 (100.0)
Physical examination performed, *n* (%)	
Otoscopy	9 (100.0)
Chest X-ray	9 (100.0)

Abbreviation: SD, standard deviation; IQR, inter-quartile range.

**Table 3:** Changes in hair-related parameters before and after HBOT.

Parameters	Before HBOT (Mean ± SD)	After HBOT (Mean ± SD)	t	*p*-Value	Cohen’s d
Overall Score (point)	73.11 ± 4.76	75.44 ± 3.88	1.871	0.098	0.624
Follicle Density (counts/cm^2^)	61.33 ± 13.00	66.78 ± 6.32	1.342	0.216	0.447
Hair per Follicle (counts)	1.24 ± 0.03	1.33 ± 0.14	1.860	0.099	0.620
Hair Volume (%)	24.89 ± 3.63	27.69 ± 4.87	1.880	0.097	0.627
Thickness (mm)	0.18 ± 0.08	0.10 ± 0.02	3.278	0.011	1.093

Abbreviation: HBOT, hyperbaric oxygen therapy; SD, standard deviation.

## Data Availability

The datasets generated and/or analyzed during the current study are not publicly available due to restrictions related to patient privacy and ethical approvals. Anonymized data may be made available from the corresponding author upon reasonable request.
